# Which risk understandings can be derived from the current disharmonized regulation of complementary and alternative medicine in Europe?

**DOI:** 10.1186/s12906-017-2073-9

**Published:** 2018-01-10

**Authors:** Solveig Wiesener, Anita Salamonsen, Vinjar Fønnebø

**Affiliations:** 1 The National Research Center in Complementary and Alternative Medicine (NAFKAM), Faculty of Health Sciences, Department of Community Medicine, UiT The Arctic University of Norway, 9037 Tromsø, Norway; 2 Regional Centre for Child and Youth Mental Health and Child Welfare (RKBU North), Faculty of Health Sciences, UiT The Arctic University of Norway, 9037 Tromsø, Norway

**Keywords:** Risk understanding, Regulation, Risk governance, Risk perception, Health governance, Complementary therapies, Alternative medicine, Traditional medicine, Patient safety, Europe

## Abstract

**Background:**

Many European citizens are seeking complementary and alternative medicine (CAM). These treatments are regulated very differently in the EU/EFTA countries. This may demonstrate differences in how risk associated with the use of CAM is perceived. Since most CAM treatments are practiced fairly similarly across Europe, differing risk understandings may influence patient safety for European CAM users. The overall aim of this article is thus to contribute to an overview and awareness of possible differing risk understandings in the field of CAM at a policymaking/structural level in Europe.

**Methods:**

The study is a re-analysis of data collected in the CAMbrella EU FP7 document and interview study on the regulation of CAM in 39 European countries.

The 12 CAM modalities included in the CAMbrella study were ranked with regard to assumed risk potential depending on the number of countries limiting its practice to regulated professions. The 39 countries were ranked according to how many of the included CAM modalities they limit to be practiced by regulated professions.

**Results:**

Twelve of 39 countries generally understand the included CAM treatments to represent “high risk”, 20 countries “low risk”, while the remaining 7 countries understand CAM treatments as carrying “very little or no risk”. The CAM modalities seen as carrying a risk high enough to warrant professional regulation in the highest number of countries are chiropractic, acupuncture, massage, homeopathy and osteopathy.

The countries understanding most of the CAM modalities in the study as potentially high-risk treatments are with two exceptions (Portugal and Belgium) all concentrated in the southeastern region of Europe.

**Conclusion:**

The variation in regulation of CAM may represent a substantial lack of common risk understandings between health policymakers in Europe. We think the discrepancies in regulation are to a considerable degree also based on factors unrelated to patient risk. We argue that it is important for patient safety that policy makers across Europe address this confusing situation. This could be done by applying the WHO patient safety definitions and EU’s policy to facilitate access to “*safe and high-quality healthcare*”, and regulate CAM accordingly.

## Background

Many European citizens are seeking a broader spectrum of treatment modalities [1–4]. This includes complementary and alternative medicine (CAM), also when offered outside their national health care system. Users of CAM mostly perceive CAM products and treatments as safe, while health care professionals often express a strong opinion that CAM treatments put patients at risk [5–7].

The World Health Organization (WHO) and the European Union (EU) have both emphasized that the first “rule” of health care is to “not harm” the patient. The WHO patient safety programme states that *“the simplest definition of patient safety is the prevention of errors and adverse effects to patients associated with health care”* [8]. This understanding underpins all regulation of health care, both of professionals and products, and is taken for granted in the 2011/24/EU Directive “*on the application of patients’ rights in cross-border healthcare*”. This directive aims to facilitate access to “*safe and high-quality healthcare*” for European citizens [9], and patients need to be confident that the treatments offered fulfill these conditions.

Regulation of health services is a risk governance tool for health authorities. The European Union (EU) assumes that a “*safe and high quality healthcare*” is dependent on a harmonized level of legislation and regulation [9]. The regulation of health care services is not included in the EU Treaty [10] or the 2006/123/EC directive on services in the internal market [11]. The EU Commission has, however, passed the Information document “*Conclusions on Common values and principles in European Union Health Systems*” to support the EU Member states in their regulation of health care services [12]. The Commission expects that a common set of operating and regulation principles will strengthen “*Quality, Safety, Patient involvement and Redress*” [12]. Further, the EU Commission emphasizes that regulation of health care must include standardization of treatments and education, recognizable and transparent legislation in all countries, standardized clinical and professional training, together with involvement of patients in their treatment. From a patient safety perspective, the EU Commission promotes regulation of health care as an important governance tool to reduce risk and facilitate high quality health care services for all European citizens.

A large study [13] recently documented that “*CAM in Europe is not regulated in accordance with current theory dealing with risk governance, risk regulation and patient safety.*” In addition, the regulation does not seem to be based on consensual scientific knowledge on effects and risks in CAM practices, efficient risk management in health care governance or systematic reports of harmful events [5, 13–15]. As a result of this apparent lack of common risk understanding, CAM is regulated “*either as conventional, complementary or alternative medicine, or not regulated at all*” [13, 16, 17]. Since the treatments themselves are practiced fairly similarly across Europe, this possible differing risk understandings could influence patient safety for European CAM users.

The overall aim of this article is thus to contribute to an overview and awareness of possible differing risk understandings in the field of CAM at a policymaking/structural level in Europe.

The specific research question is:

Which risk understandings may be derived from the current regulation of CAM in 39 European countries?

## Methods

This study is a re-analysis of data collected in the CAMbrella EU FP7 document and interview study on CAM regulation in Europe [13, 16, 17]. Data dealing with the general legal and regulatory status of CAM on the first and second national legal level, including 12 specific CAM treatment modalities and CAM providers/professions, were collected from the European Union (EU) and 39 European countries. The included CAM modalities were acupuncture, anthroposophic medicine, ayurvedic medicine, chiropractic, herbal medicine/phytotherapy, homeopathy, massage, naprapathy, naturopathy, neural therapy, osteopathy and Traditional Chinese Medicine (TCM).

The 12 CAM modalities included in the CAMbrella study were in this study ranked with regard to assumed risk potential depending on the number of countries limiting practice of CAM modalities to regulated professions. We ranked the 39 countries according to how many CAM modalities they limit to regulated professions. We have arbitrarily classified countries limiting 5 or more of 12 CAM modalities to regulated professions as seeing CAM as high-risk treatment approaches, and regulating 1–4 as seeing CAM as carrying a potential low patient risk. If a country has not limited any modality to a regulated profession, we classify the country as seeing CAM with very little or no risk.

## Results

**Fig. 1:**
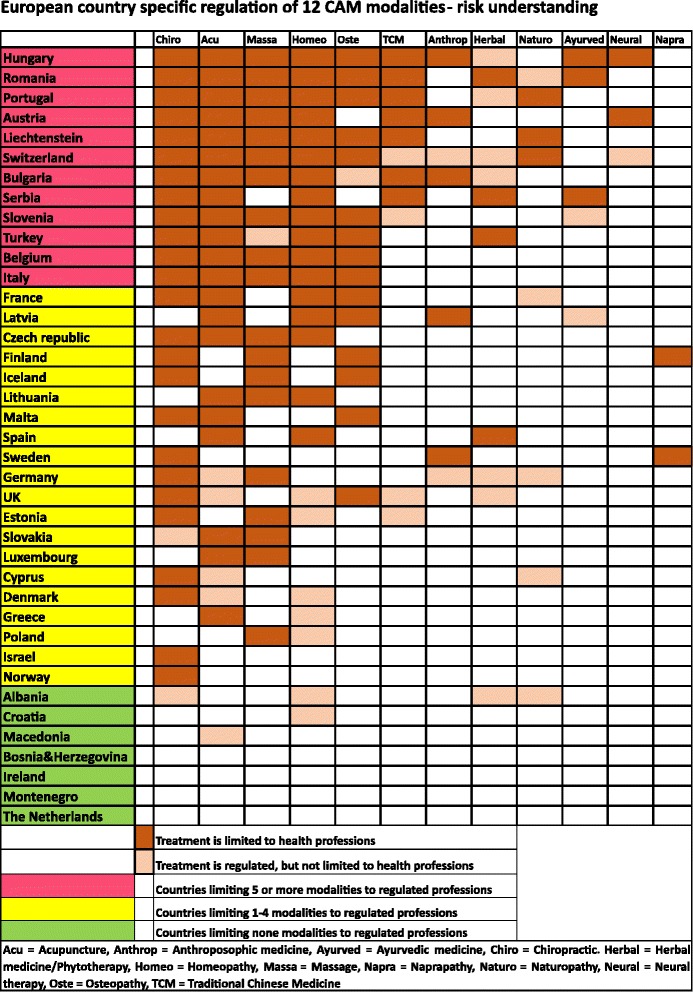
European country specific regulation of 12 CAM modalities-risk understanding

We found that 12 countries understand the included CAM treatments as carrying a high risk, 20 understand the CAM treatments to have low risk, while the remaining 7 countries generally understand the CAM treatments to be with very little or no risk (Fig. 1). We also found that countries understanding CAM to be associated with low, very little or no risk, only regulate modalities that are the most commonly regulated in the countries with a high-risk understanding. There are, however, two country exceptions worth noting; Finland and Sweden also regulate naprapaths.

The CAM modalities seen as carrying a risk high enough to warrant professional regulation in the highest number of countries are chiropractic, acupuncture, massage, homeopathy and osteopathy. The “manual” therapies (naprapathy, massage, osteopathy and chiropractics) are almost exclusively regulated as specific health professions with protected titles in the countries that have chosen to regulate.

**Fig. 2:**
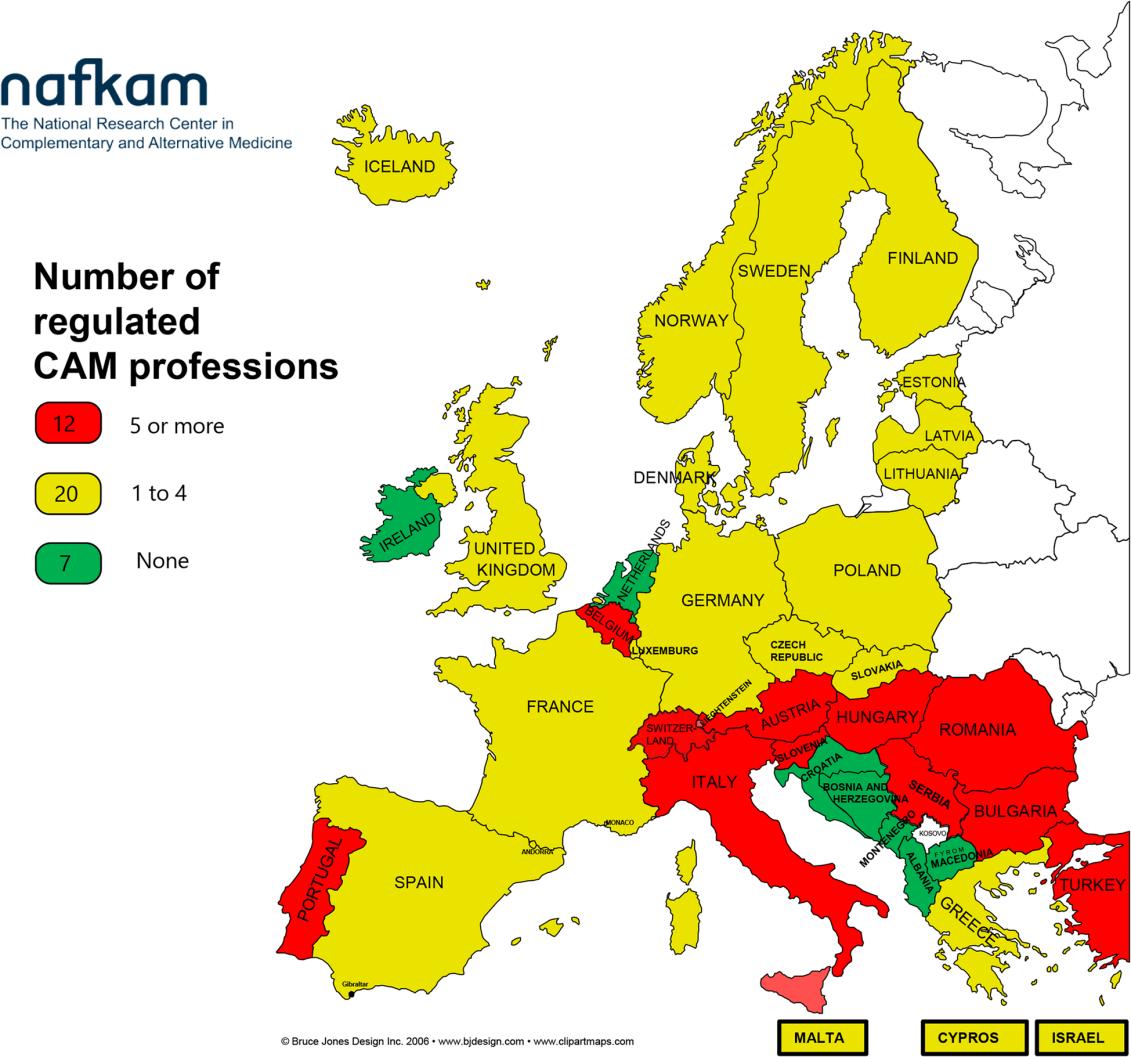
Risk understanding-number of regulated CAM professions. “Risk understanding-number of regulated CAM professions” depicted in Figure 2 has been developed by the authors and has not been published before. The map is developed on the basis of an uncolored clip-art map of the European countries, retrieved from: “Bruce Jones design Inc. 2006-www.bjdesign.com”. The clip-art map was purchased first time for use by NAFKAM in the CAMbrella project FP7-HEALTH-2009, GA No.241951. NAFKAM allows the authors to develop maps using this purchased clip-art

The countries that seem to understand CAM treatment to be associated with a potentially high patient risk are not randomly distributed across Europe. With two exceptions (Portugal and Belgium), they are all concentrated in the southeastern region of Europe (Fig. 2).

## Discussion

This study demonstrates that the regulation of CAM indicates the differences in how the European health authorities may perceive risk associated with the use of CAM.

We do not extrapolate our findings beyond the 39 countries studied, and there is therefore no selection bias in this study with regard to countries. The procedure followed to gain overview of the regulatory situation was comprehensive [13, 16, 17], and the regulation in each country is constantly being updated [18]. To our information, no one has successfully contested our descriptions, and the information presented is therefore likely to be unbiased.

To our knowledge, no one has previously described international CAM regulation in a risk perspective focusing on risk understanding.

In the introduction to the 2011/24/EU Directive “on the application of patients’ rights in cross-border healthcare”, the European Council assumes that “safe and high-quality healthcare” is in place when declaring that there “*is a set of operating principles that are shared by health systems throughout the Union*” (Directive 2011/24/EU Directive- introduction note 5) [9]. The most common used “operating principles” in providing safe and high-quality healthcare are to regulate the professional requirements of health providers and standardization of treatments [13]. Baldwin claims that *“..regulation is often thought of as an activity that restricts behavior and prevents the occurrence of certain undesirable activities”* [19].

Previous studies show that conventional healthcare is regulated quite similarly across Europe. Core health professionals are defined as “Sectorial professions” benefiting from automatic recognition on the basis of harmonization of minimum training conditions (doctors, nurses, midwives, pharmacists and dentists) [13, 20]. High quality and safety are perceived as established through appropriate research and sound professional standards, at the same time as attractive treatment options across borders are available [21].

We would have expected to find similar sets of operating principles in the regulation of CAM. The most regulated CAM modality in Europe is chiropractic. Does this mean that chiropractic treatment is associated with such a high patient risk that 25 European countries see strong regulation as necessary, or could the regulation be based on other criteria as well? A closer look at the process of regulating chiropractors in Norway in 1988 sheds light on this possibility [22]. The decision to regulate was based on a 1985 governmental report. The arguments for giving chiropractors authorization and a protected title were to secure the educational requirements for practicing chiropractic. The politicians stated that regulation of chiropractors would strengthen the profession and lead to recognition of the chiropractors as health professionals, nothing about risk [23].

Taking acupuncture regulation as an additional example, we found no consistent pattern of risk understanding. Acupuncture treatment is the second most regulated CAM modality in Europe, but only 3 countries have established “acupuncturist” as a profession. In Hungary 3–5 years of acupuncture education/training at a university level is required in addition to being a medical doctor. In Norway acupuncture can be legally practiced without any educational requirements whatsoever. Based on our classification, acupuncture is understood to be a high-risk treatment in Hungary, while Norwegian health authorities seem to understand the same treatment to be without risk. The “high-risk” regulation in Hungary could possibly be based on registered adverse events, number of harmed patients and claims, combined with risk or effect research. If so, why do they not take into account this risk information in the “no-risk” regulation in Norway?

An interesting example of risk understanding linked to the importance of professional knowledge is the regulation of homeopathy in Belgium. The “Belgian Healthcare Knowledge Centre” stated in a public report in 2011 that due to safety reasons, homeopathy should be restricted to physicians, dentists and veterinary doctors [24]. Consequently, “*The Belgian Council of Ministers passed new regulation on homeopathy in 2013, made official by a Royal Decree published 12 May 2014 by the Ministry of Health”* [18]. This decision is consistent with Belgian regulation of other common CAM practices (Fig. 1 European country specific regulation of 12 CAM modalities-risk understanding). We hypothesize that the risk perception of the Belgian health authorities is of an “indirect” nature.

### Regulation of CAM- a decision model

**Fig. 3:**
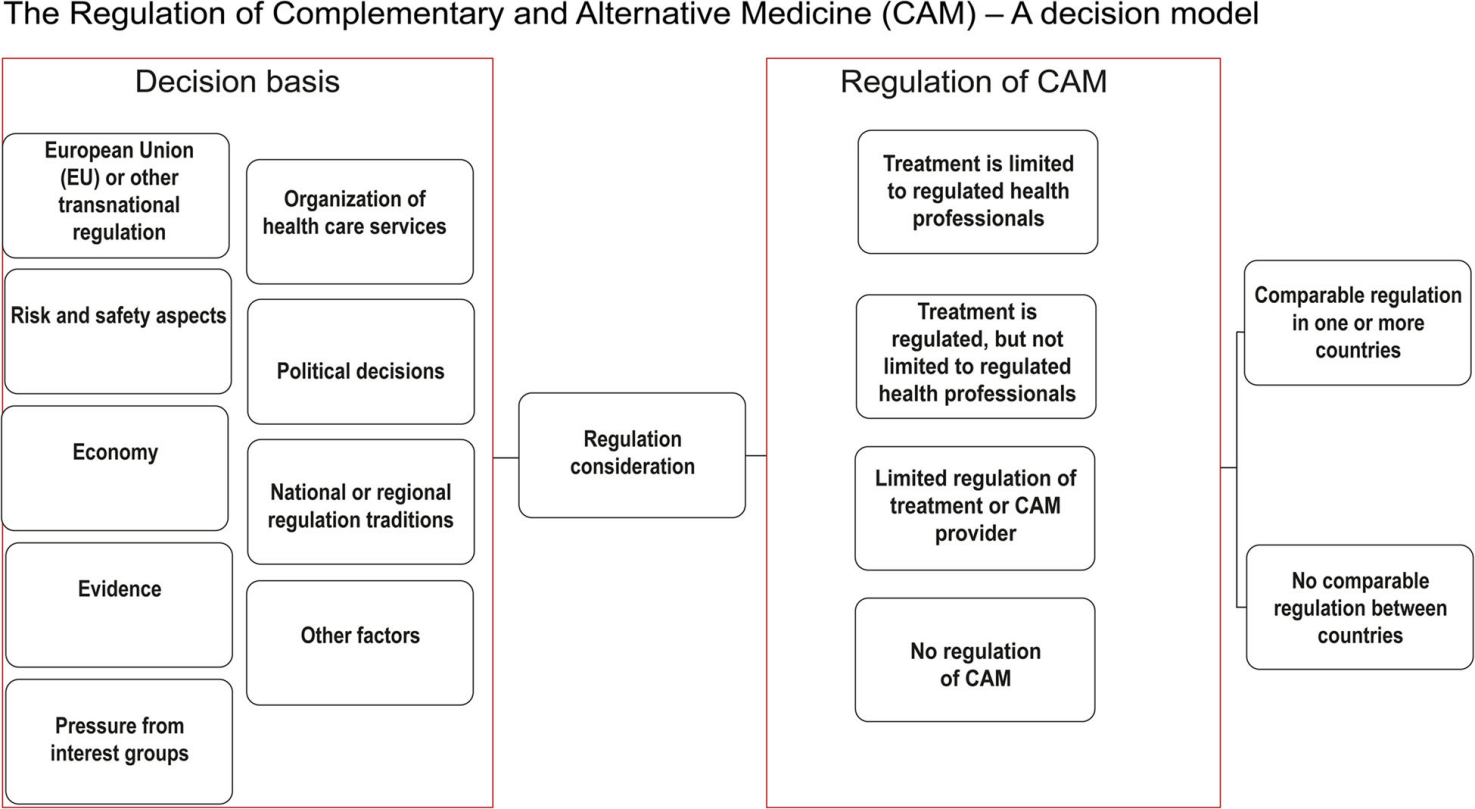
Regulation of CAM-a decision model

Risk associated with health care is often separated into *direct risk* caused by the treatment itself or *indirect risk* related to adverse effects of the treatment context [15]. The discrepancies in regulatory practice between countries are, in our view, only explicable if factors other than direct or indirect risk are taken into consideration. Based on the regulation status across Europe, as well as established risk and health management research and legislation, we have developed a theoretical decision model we think more truly reflects the political process relevant for the regulation of CAM (Fig. 3).

The model includes multiple factors that come into play when a country considers the regulation of CAM, and the chosen level of regulation will reflect the factors given most importance.

The map showing CAM regulation presented in this article (Fig. 2) illustrates the potential importance of national and/or regional traditions. This was already suggested by Gerd Ersdal 12 years ago [25]. She divided European countries into “all-regulated” and “semi-regulated” systems of CAM regulation, and suggested that this geographical megapattern seemed to indicate that in the middle and south of Europe CAM regulation followed the principle: “if regulated-it is allowed”. In northern Europe the principle “if not forbidden by law- it is allowed” seemed to dominate. But even within these regions the regulations of CAM varies substantially. It is difficult to understand the basis for why they have regulated the way they have done.

## Conclusion

The variation in regulation of CAM may represent a substantial lack of common risk understandings between health policymakers in Europe. We think the discrepancies in regulation are to a considerable degree also based on factors unrelated to patient risk. We argue that it is important for patient safety that policy makers across Europe address this confusing situation. This could be done by applying the WHO patient safety definitions and EU’s policy to facilitate access to “*safe and high-quality healthcare*”, and regulate CAM accordingly.

## Abbreviations

CAM: Complementary and alternative medicine
EFTA: The European Free Trade Association
EU: The European Union
WHO: The World Health Organization
